# The Prevalence and Classification of the Cystoduodenal Ligament

**DOI:** 10.1155/2015/742621

**Published:** 2015-08-12

**Authors:** J. O. Ashaolu, J. Olayinka, V. O. Ukwenya

**Affiliations:** ^1^Department of Anatomy, Faculty of Basic Medical Sciences, College of Health Sciences, Bowen University, Iwo, Osun State 284, Nigeria; ^2^Department of Anatomy, Faculty of Basic Medical Sciences, College of Medicine, Ekiti State University, Ado-Ekiti, Ekiti State 5363, Nigeria

## Abstract

Variant patterns of peritoneal folds could be formed due to the complex nature of the embryology of the peritoneum and the gastrointestinal tract. When uncommon peritoneal folds are formed, they could influence aberrant formation of surrounding gastrointestinal structures and create spaces that may harbour peritoneal fluids in cases of infection or malignant tumor. One of such variant peritoneal folds is the cystoduodenal ligament which is a doubled peritoneal membrane attaching the gallbladder to the duodenum. Yet no study was found that had reported the frequency of occurrence of the cystoduodenal ligament. The current study determined the prevalence of the cystoduodenal ligament in forty adult cadavers. The ligament was reported in 35% of cases. The ligament was further classified as types I and II. Type I cystoduodenal ligament was attached partially to the gallbladder (neck and proximal part of body) while type II was attached to the entire extent of the gallbladder. Type I occurrence was found in 44% and type II was found in 56% of the occasions of cystoduodenal ligament formation. It is concluded that the cystoduodenal ligament could be commonly found, it possesses important vascular structures, and it could affect the shape of the gallbladder. Surgeons, radiologists, and anatomists should be kept abreast of these findings.

## 1. Introduction

It is important to take cognizance of the possibilities of encountering variant peritoneal folds when assessing the abdominal region, either radiologically, surgically, or in cadaveric demonstrations. Such peritoneal formations may potentiate the kinking of intestinal structures, leading to intestinal necrosis or increased tendency of intestinal herniation [1]. They could also contain important neurovascular structures [1–3]. One of such folds is the cystoduodenal ligament which binds the gallbladder to the superior part of the duodenum. The cystoduodenal ligament is a variant ligament of the lesser omentum different from the commonly known hepatoduodenal and hepatogastric ligaments [1, 4, 5]. The hepatogastric ligament joins the visceral surface of the liver to the lesser curvature of the stomach while the hepatoduodenal ligament joins the visceral surface of the liver to the superior aspect of the duodenum [5]. The cystoduodenal ligament is a double-layered membranous structure [1, 4, 5] which has been reported in few instances and generally considered an occasionally occuring structure, being previously reported mainly in cadaveric bodies [1, 4, 5]. The lesser omentum is a derivative of the ventral mesogastrium and it is also a landmark structure in the delineation of peritoneal effusion [1]. However, the cystoduodenal ligament has been described as a guard to the entrance of the omental foramen [1]. But, till date, there is paucity of information as regards the frequency of occurrence of the cystoduodenal ligament. This current work seeks to establish the prevalence of the cystoduodenal ligament in forty cadavers.

## 2. Materials and Methods

The study was conducted on forty cadavers (thirty-one males and nine females) in the Department of Anatomy, Bowen University, Osun State, Nigeria. This study was conducted between February 2011 and October 2014. The use of cadavers was in line with the Nigeria Anatomical Act. Departmental approval was sought from the head of department of the involved institution. The ages of the cadavers were not retrievable but they were presumably adults.

The abdominal cavity was carefully dissected, and then the supracolic region was painstakingly exposed. The visceral surface of the liver was upwardly reflected to subject the cystoduodenal ligament to qualitative and quantitative examination. Qualitative analysis was done by physical visual examination while quantitative measurements were performed with the digital vernier caliper.

The presence of the cystoduodenal ligaments was confirmed when ligaments were seen being superiorly attached to the inferior border of the gallbladder, either completely or partially; and they are distally attached to the duodenum. The cystoduodenal ligament was carefully dissected to record the possible existence of the cystic artery, vein, or duct. The upper, middle, and lower width of the cystoduodenal ligament were measured.

The cystoduodenal ligament was differentiated from peritoneal adhesion by tracing the two membranes that formed it superiorly and inferiorly around the gallbladder and duodenum. The continuity of the two membranes of the cystoduodenal ligament with visceral peritoneum of the gallbladder and the duodenum is a confirmation that they are not mere peritoneal adhesions.

## 3. Results

**Figure 1:**
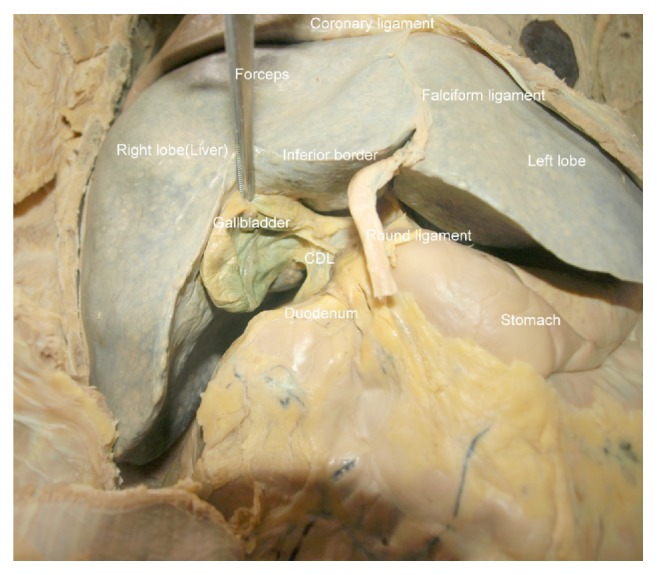
Showing type I cystoduodenal ligament (CDL) attaching superiorly to the entire extent of the inferior aspect of the gallbladder and inferiorly to the superior aspect of the duodenum.

**Figure 2:**
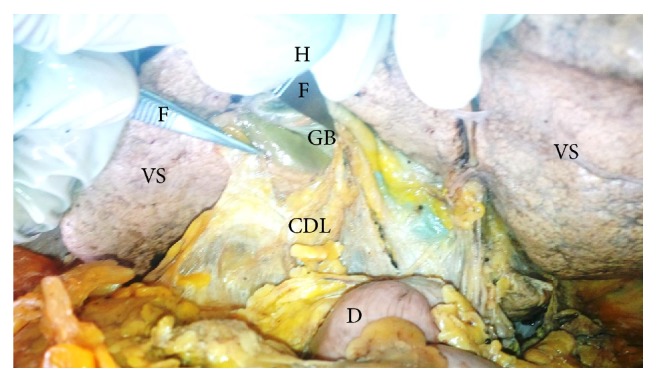
Showing type II cystoduodenal ligament attaching superiorly to the entire extent of the inferior aspect of the gallbladder and inferiorly to the superior aspect of the duodenum. CDL: cystoduodenal ligament, GB: gallbladder, VS: visceral surface of liver, D: superior part of duodenum, F: forceps, and H: hand of demonstrator.

This current study reports 35% (fourteen out of forty) occurrence of the cystoduodenal ligament (Figures 1 and 2). The ligament was present in 42% of males (thirteen out of thirty-one) and 11% (one out of nine) of females. The cystoduodenal ligaments that were partially attached to the gallbladder were classified as type I (Figure 1) while those that were completely attached to the gallbladder were recorded as type II (Figure 2). Type I occurrence was found in 44% (six out of fourteen) and type II was found in 56% (eight out of fourteen) of the occasions of cystoduodenal ligament formation. Inferiorly, the cystoduodenal ligament was attached to either the superior part of the duodenum and/or the descending part of the duodenum. The ligament was measured after the liver visceral surface was reflected upwards. The upper width was 4 cm and the middle width was 5 cm while the lower width was 5 cm. In all occasions, the ligament retained a potential space named the retrocystoduodenal recess behind it. Also, the omental foramen was potent in all the dissected cadavers. Doubly folded gallbladder was found in two instances (5%) and they were found to be associated with type I cystoduodenal ligament only. Anomalous duodenum was not found in any instance. The contents of the cystoduodenal ligament were either the cystic artery, vein, or cystic duct in 68% of occassions. The cystic artery, duct, and vein existed within the hepatodeuodenal ligament in the cases where the cystoduodenal ligaments were not present.

## 4. Discussion

The current study is the first to demonstrate the prevalence of the cystoduodenal ligament. The study demonstrated that the cystoduodenal ligament is more prevalent (35%) than it was ocassionally expected [3]. This study also provided the structural classification of the cystoduodenal ligament. Cases of variant peritoneal bands such as cystoduodenal, cystocolic, or cystogastrocolic extension of the lesser omentum have been reported [1, 3–9]. Such bands have redefined the conventional margins of the omental foramen and sometimes caused its obstruction [8–10]. Two occurences of cystoduodenal ligament found in Nigerian cadavers were described by Ashaolu et al. in 2011 [1]; another was reported by the same author in 2012 [4]. Sharma et al. [5] reported another case in India in 2013. The combined statistics of a number of observers showed that uncommon peritoneal bands occurred in about 22% of cases [7].

It was ensured in the current work that the cystoduodenal ligament was differentiated from peritoneal adhesion which usually occurs due to peritonitis. The formation of the cystoduodenal ligament would be clinically important if it disrupts the function(s) of the surrounding structures [3]. The contents of the cystoduodenal ligaments, that is, the cystic artery, vein, or cystic duct, which occurred in 68% of occasions are very important structures. However, iatrogenic damage of the contents during surgical ligation of the ligament could be prevented if surgeons are aware of the anatomy of the ligament.

The cystoduodenal ligament was associated with anomalous gallbladder in 5% of all cases studied and only type I formation was involved. This supports earlier finding that peritoneal folds could disrupt gallbladder structure and emptying [11], while a chronic disruption could affect the delivery of bile to the gastrointestinal tract and thus impede digestion. Type I cystoduodenal ligament formation could produce differential possibilities of gallbadder shapes on sonography such as phrygian cap, dessquamated gallbladder mucosa, or extension of the spiral valve of Heister into the gallbladder neck region or polyploid cholesterolosis [12]. Type II formation could result into gallbladder thickening, which was always taken as an indication for gastrointestinal pathology [13]. The gallbladder may also have a mesentery attaching it to the visceral surface of the liver which could sometimes cause its volvulus [11, 14]. Abnormal peritoneal folds around the gall bladder might restrict its expansion during its filling processes [11]. The walls of the gall bladder associated with variant peritoneal fold were reported to be thicker than those in normal cases and the reason was attributed probably to resistance offered by the peritoneal fold during the distension of the gallbladder [11].

Congenital bands have been reported to cause contriction or obstruction of duodenum or jejunum [15, 16]; however, this current study did not report any form of duodenal constriction.

It should be noted that, commonly, variant peritoneal fold formations result from abnormal gastrointestinal rotation and they sometimes occur alongside gastrointestinal anomalies. In the case reported by Ashaolu et al. [1, 4], the cystoduodenal ligament was associated with abnormal intestinal formation where there were absent ascending colon, thin transverse colon, and redundant sigmoid colon. In the case reported by Sharma et al. [5] it was part of many other variant ligaments described; in that case, there was formation of an aberrant ligament that connects the inferior surface of the right lobe of liver to both the duodenum and the transverse colon, the distal half of the transverse mesocolon was arched, and there was aberrant mesentery of descending colon and redundant formation of the sigmoid colon.

The mode of origin of variant peritoneal folds has been attributed to errors in development, previous inflammation (peritonitis), and mechanical traction by the gut [3]. It has been postulated that the embryological origin of the cystoduodenal ligament is from the abnormal fusion of ventral and dorsal mesogastria [1, 11]. Since the gallbladder alongside the liver develops within the ventral mesentry, we currently propose that the formation of the cystoduodenal ligament might just be as a result of persistency of the part of the ventral mesentery that connected the primitive gallbladder to the developing duodenum.

The cystoduodenal ligament formation may potentiate the kinking of intestinal structures, leading to intestinal necrosis or increased tendency of intestinal herniation [1, 4, 5]. They could also contain important neurovascular structures. When the cystoduodenal ligament is present, retrocystoduodenal recess herniation or kinking may be more common. Although the cystoduodenal ligament appears as a relative protector of the omental foramen, it could compound omental foramen herniation when it occurs [1, 4, 5].

It is therefore important for surgeons, anatomists, and radiologists to be aware of the dominance of the ligament.
